# Proximal medial patellar restraints and their surgical reconstruction

**DOI:** 10.1186/s10195-019-0525-3

**Published:** 2019-03-26

**Authors:** Paolo Ferrua, Bradley M. Kruckeberg, Stefano Pasqualotto, Massimo Berruto, Pietro Randelli, Elizabeth A. Arendt

**Affiliations:** 1 I clinica ortopedica ASST Gaetano Pini-CTO Gaetano Pini, Via Quadronno, 25, 20122 Milan, Italy; 2 Department of Orthopedic Surgery, Mayo Clinic, 200 1st Street SW, Rochester, MN 55905 USA; 3 Department of Orthopaedic Surgery, University of Minnesota, 2450 Riverside Avenue South, Suite R200, Minneapolis, MN 55454 USA

**Keywords:** Medial patellar femoral ligament, Proximal medial patellar restraints, Patellar instability

## Abstract

Reconstruction of the medial patellofemoral ligament (MPFL) has been increasing as a surgical solution for treatment of recurrent lateral patellofemoral dislocation. Recent attention has been given to fibers extending from the femur to the quadriceps tendon, proximal to the MPFL, termed the medial quadriceps tendon-femoral ligament. This article briefly reviews the proximal medial patellar restraints and surgical procedures for their reconstruction.

## Introduction

Knowledge of patellofemoral (PF) joint pathophysiology and kinematics continues to evolve. Several anatomic structures have been identified as playing a role in the biomechanics and pathophysiology of injury to the medial side of this joint. These structures are typically divided into dynamic and static stabilizers. Historically, the static stabilizers have been labeled the patella retinaculum, and include the medial patellofemoral ligament (MPFL) and patellotibial ligament. More recently, the medial patellotibial ligament (MPTL) and medial patellomeniscal ligament (MPML) have gained recognition in literature [1, 2], as well as the medial quadriceps tendon-femoral ligament (MQTFL) [3, 4].

With the increasing recognition of the medial patellar restraints, one can divide these ligaments into the proximal medial patellar restraints (MQTFL + MPFL) and the distal medial patellar restraints (MPTL + MPML).

Classically, PF joint stabilizing procedures have involved alteration of dynamic stabilizers, in particular the vastus medialis obliquus (VMO) muscle. Literature has shown that static stabilizers, in particular the MPFL, play an important role in patellofemoral biomechanics [5–7], showing correlations with injury when measured using advanced radiographic techniques including magnetic resonance imaging (MRI) [8–10] or stress x-rays [11], and surgically after injury [12–14]. As a result, current procedures involve static stabilizers of the patellofemoral joint, especially the medial patellofemoral ligament (MPFL) and lateral retinacular complex. Reconstruction of the MPFL has become more common as a surgical solution for treatment of recurrent lateral patellofemoral dislocation [15].

This article briefly reviews the proximal medial patellar restraints and surgical procedures for their reconstruction.

## Brief literature review

In 1948, Last et al. [16] described the medial anatomy of the knee joint, stating that strong retinacular fibers extend from the medial border of the patella “towards the medial collateral ligament.” The MPFL, though not described by name, inserted distal to, or underneath, the femoral insertion of the medial collateral ligament (MCL).

In 1979, Warren and Marshall [17] published a thorough description of the medial side of the knee that was organized into a three-layer system. The MPFL and medial retinaculum are extracapsular structures described within layer II, the same layer as the superficial MCL. The authors describe a common insertion site of the MCL, MPFL, and the adductor magnus tendon on the medial epicondyle. The medial retinacular ligaments were identified as thickening of tissue planes more than independent structures. They further state that, on the medial side of the knee, this three-layer anatomy is often intimately intertwined, with only a few places where three distinct layers could be clearly separated.

Over a decade later, in 1993, Conlan et al. [5] published a paper describing an anatomic and biomechanical study of the medial soft tissue restraints of the patellofemoral joint. In the authors’ in vitro cutting study, the contribution of the MPFL to medial static restraint ranged from 23 to 80 %.

**Fig. 1:**
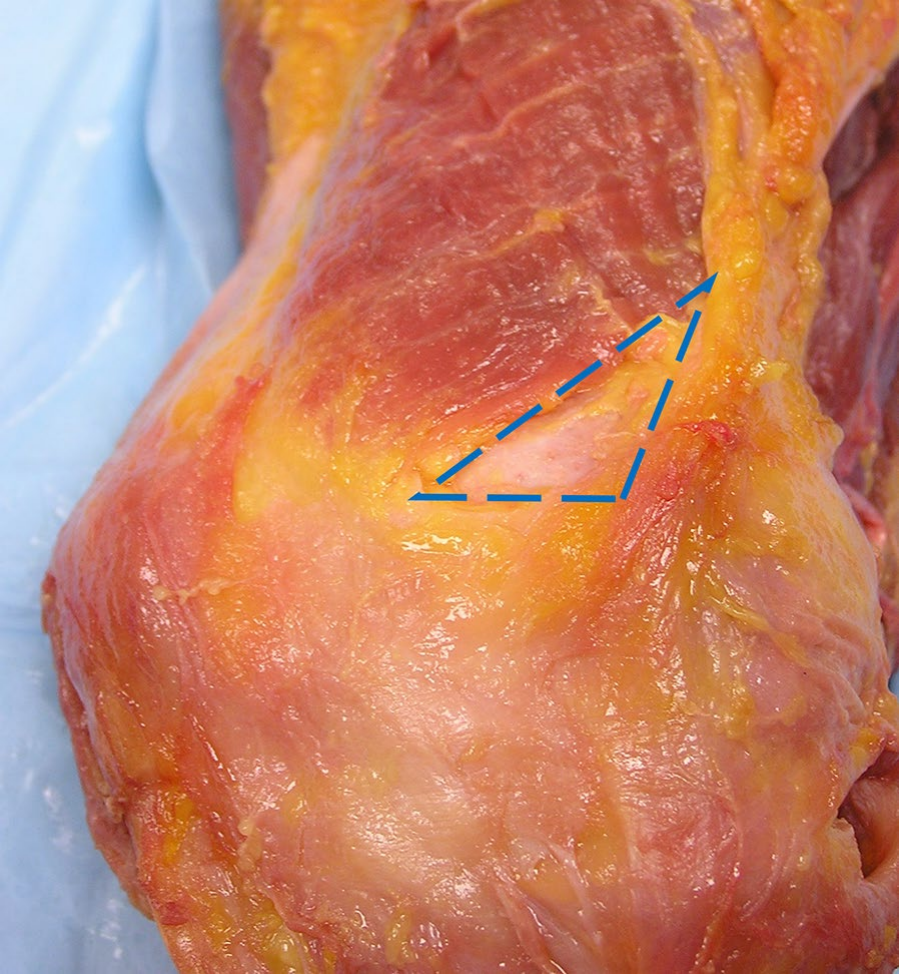
The “triangle of Tuxøe” (dotted lines) is formed by the adductor magnus tendon medially, VMO fibers superolaterally, and the superior border of the MPFL distally

A consistent anatomic feature that can be utilized to locate the MPFL is identification of the “triangle” described by Tuxøe et al. [18] and discussed by Feller et al. [19] (Fig. 1). This triangle or bare spot is formed by the adductor magnus tendon medially, VMO fibers superolaterally, and the superior border of the MPFL distally.

**Fig. 2:**
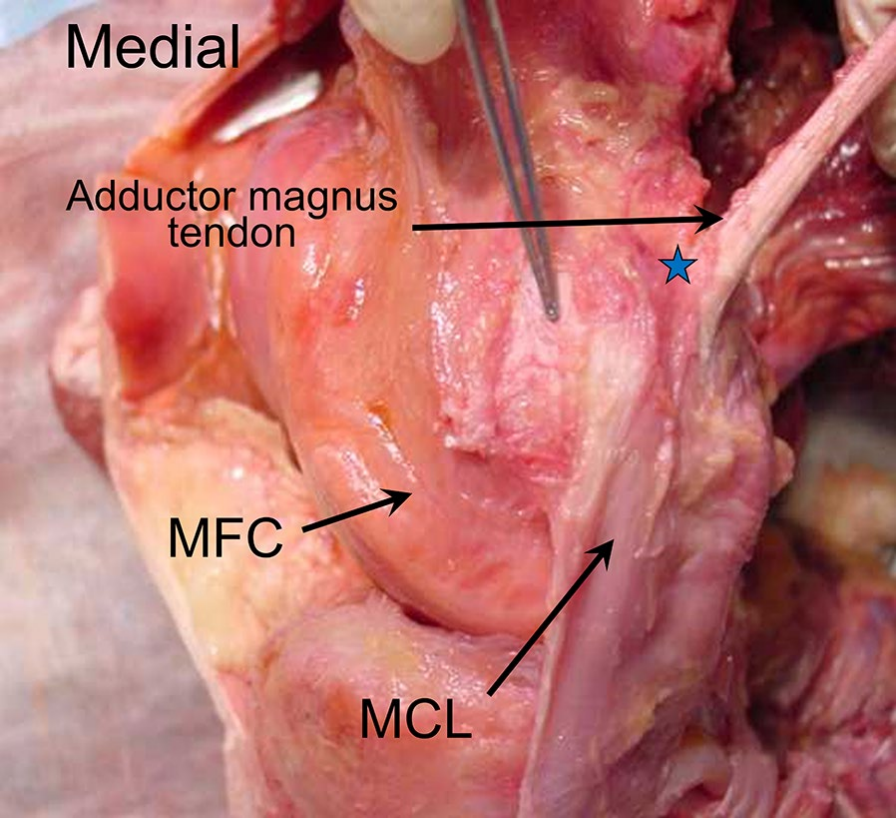
Cadaveric specimen stripped of medial soft tissue except MCL and adductor magnus tendon. Star indicates the area of the femoral insertion of the MPFL

Medial patellofemoral ligament anatomy was further defined by Smirk et al. [20], who described equally suitable sites for graft attachment during reconstruction. The femoral attachment site of the MPFL was not uniform, with the most common site being the posterior portion of the medial epicondyle, approximately 1 cm distal to the adductor tubercle (Fig. 2). This insertion site was in isolation 44 % of the time. In another 40 %, the attachment was part of a wider area including the adductor magnus tendon (12 %), the area just posterior to this (20 %), or some combination of the above (4 %). In 16 %, the MPFL was found attached to the anterior part of the medial epicondyle.

An anatomic review of the medial patellofemoral ligament by Amis et al. [21] noted the lack of consistency in literature regarding the MPFL femoral attachment, and suggested that the convergence of a number of structures and tissue layers towards the medial epicondyle makes it difficult to separate the MPFL. The authors observed that the MPFL fibers decussate as they approach the femoral attachment, and speculated that the MPFL may have two functional bands that run along its proximal and distal fibers.

Nomura et al. [22] dissected 20 knees, and reported an insertion site on the superior–posterior aspect of the medial femoral condyle, just distal to the adductor tubercle (on average 10 mm proximal and 5 mm posterior to the center of the medial epicondyle). On the patella side, VMO fibers cover approximately 35 % of the MPFL as it inserts on the proximal/medial patella. The center point of the MPFL insertion on the patella is at 27 ± 10 % from the upper end of the patella when measured longitudinally on its ventral bony surface.

In 2007, LaPrade et al. [23] reported qualitative and quantitative descriptions of the attachment sites of main medial structures of the knee. For the MPFL, they quantified the midpoint of the MPFL patella attachment as located 41.4 % of the length from the proximal tip of the patella compared with the total patella length. The attachment on the femur was on average 10.6 mm proximal and 8.8 mm posterior to the medial epicondyle, 1.9 mm anterior and 3.8 mm distal to the adductor tubercle. The average length of the MPFL was 65.2 mm [23, 24].

In 2009, Baldwin [25] described the anatomy of the MPFL based on 50 fresh or fresh-frozen anatomic specimens. His eloquent description provided rich anatomic detail of the MPFL femoral insertion. A transverse origin was identified from the bony groove between the medial epicondyle and the adductor tubercle, in addition to a second oblique desiccation originating from the proximal edge of the MCL. Several authors support that the MPFL has multiple fiber extensions to other sites in this region [18, 20, 21, 26, 27] and that it is clearly an extracapsular structure [5, 18, 20, 21, 26].

In the pediatric population, studies consistently cite the MPFL femoral origin distal to the medial distal femoral physis [28, 29].

**Fig. 3:**
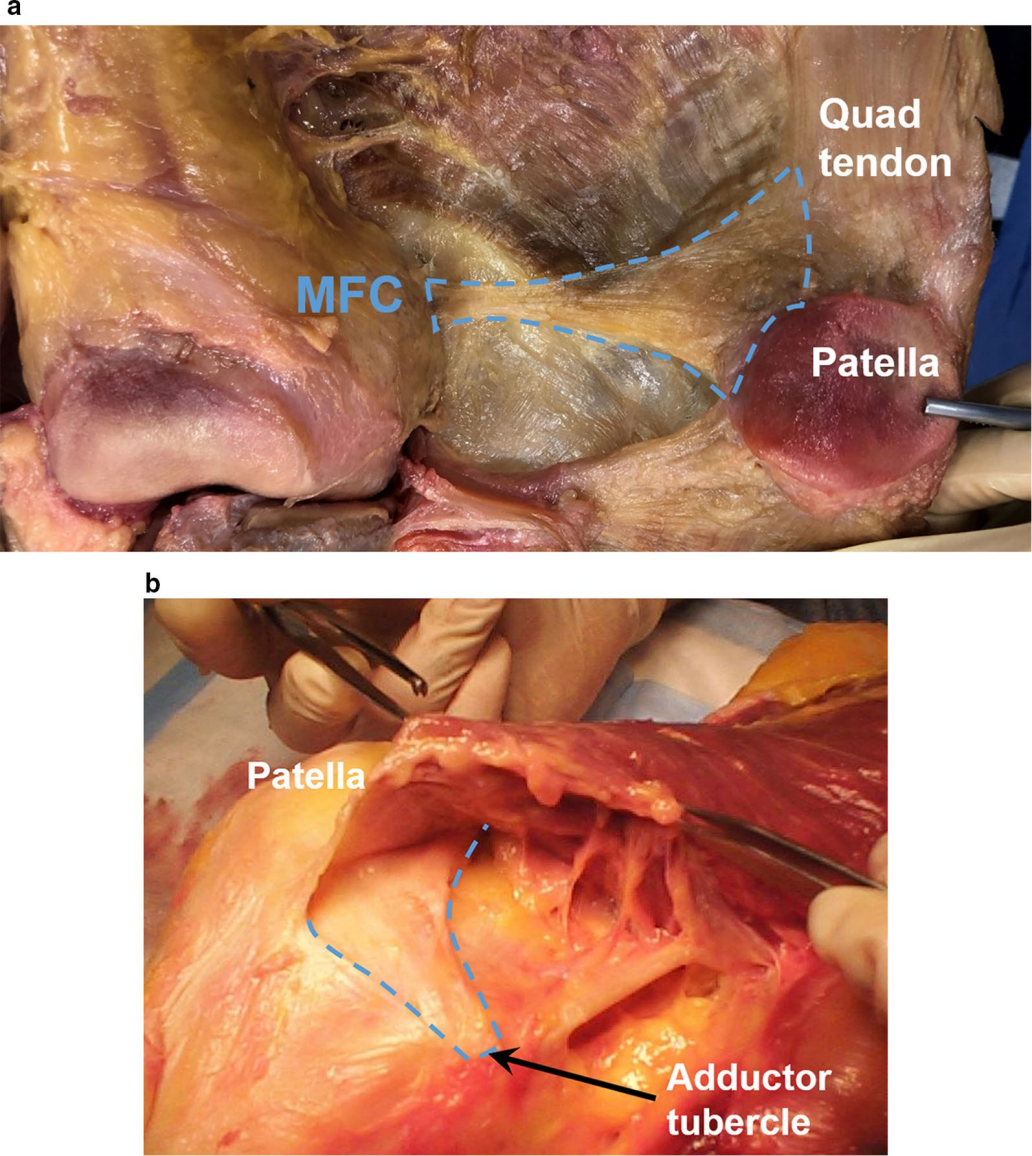
**a** Cadaveric specimen depicting the undersurface of the medial muscles and soft tissue structures. Outlined is the medial proximal patella femoral complex, which demonstrates fibers originating on the femur and extending to the superior medial patella and quadriceps complex. The superior portion of this complex has been termed the medial quadriceps tendon-femoral ligament (MQTFL). Image courtesy of Miho J. Tanaka MD, Johns Hopkins, Baltimore, MD. **b** Cadaveric specimen with the medial muscles translated superiorly. The broad proximal medial patellar restraints are depicted, fanning out from the adductor tubercle to the patella and distal quadriceps complex

Recently, attention has returned to the fibers extending from the femur to the quadriceps tendon that have been previously described [20, 21]. These fibers, proximal to the MPFL, have been termed the medial quadriceps tendon-femoral ligament (MQTFL) (Fig. 3) [3, 4]. Despite the presence of proximal fibers, their correlation with injury [10] and the added role of reconstruction of these fibers for patella stabilization is debated.

**Fig. 4:**
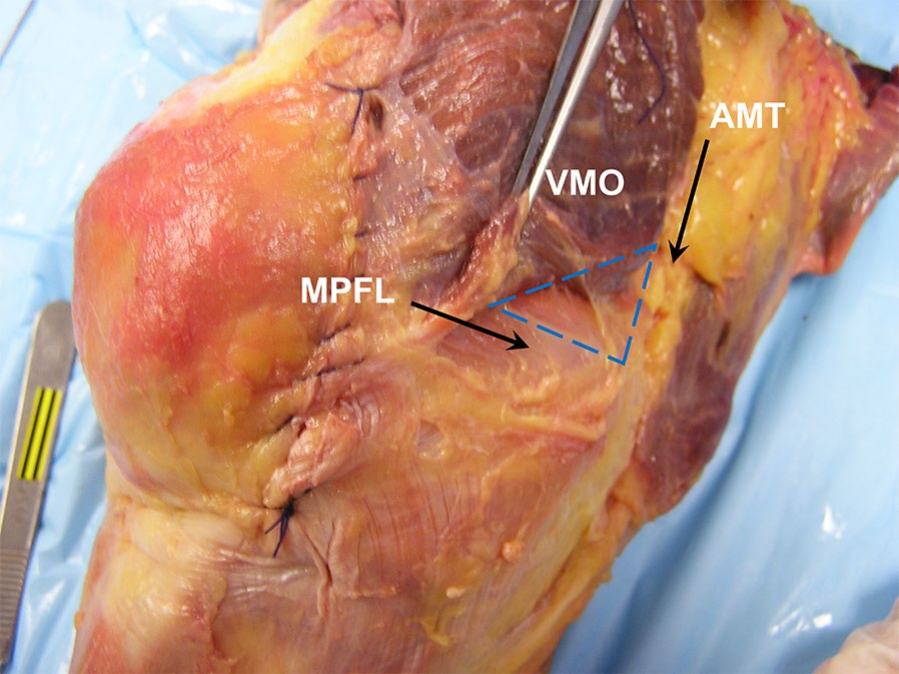
Cadaveric specimen where the VMO fibers nearly completely cover the MPFL and one has to “lift up” the VMO to expose the MPFL ligament. The “triangle of Tuxøe” is visible. In most cases of lateral patellar dislocation, the VMO is dysplastic and the MPFL is “uncovered” to varying degrees

**Fig. 5:**
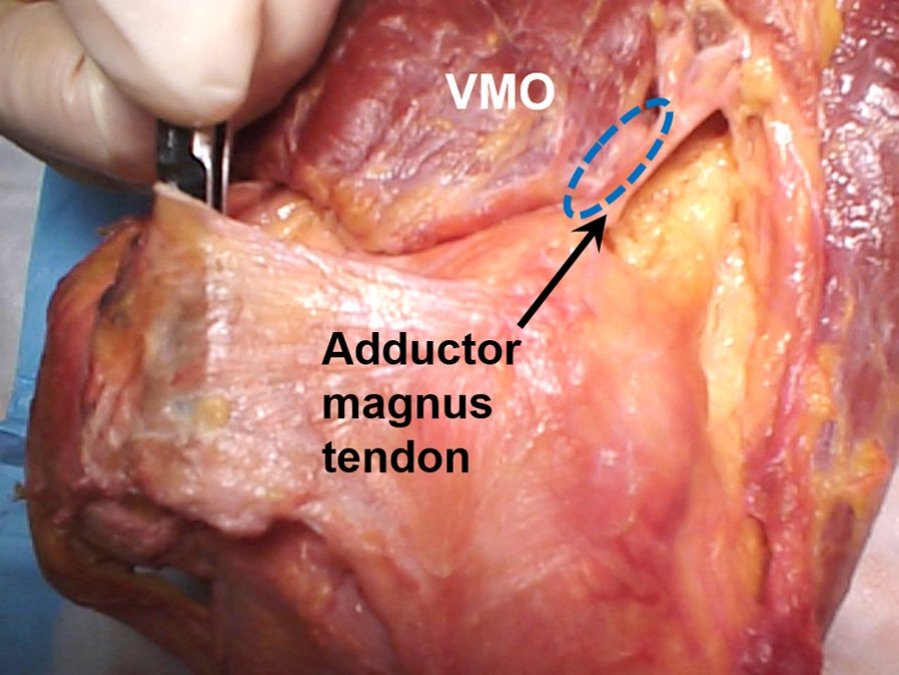
Cadaveric specimen depicting the VMO fibers attaching along the adductor magnus (AM) tendon; in traumatic lateral patellar dislocation, these fibers can tear away from the AM tendon, potentially creating a more vertical pull of the VMO fibers

**Fig. 6:**
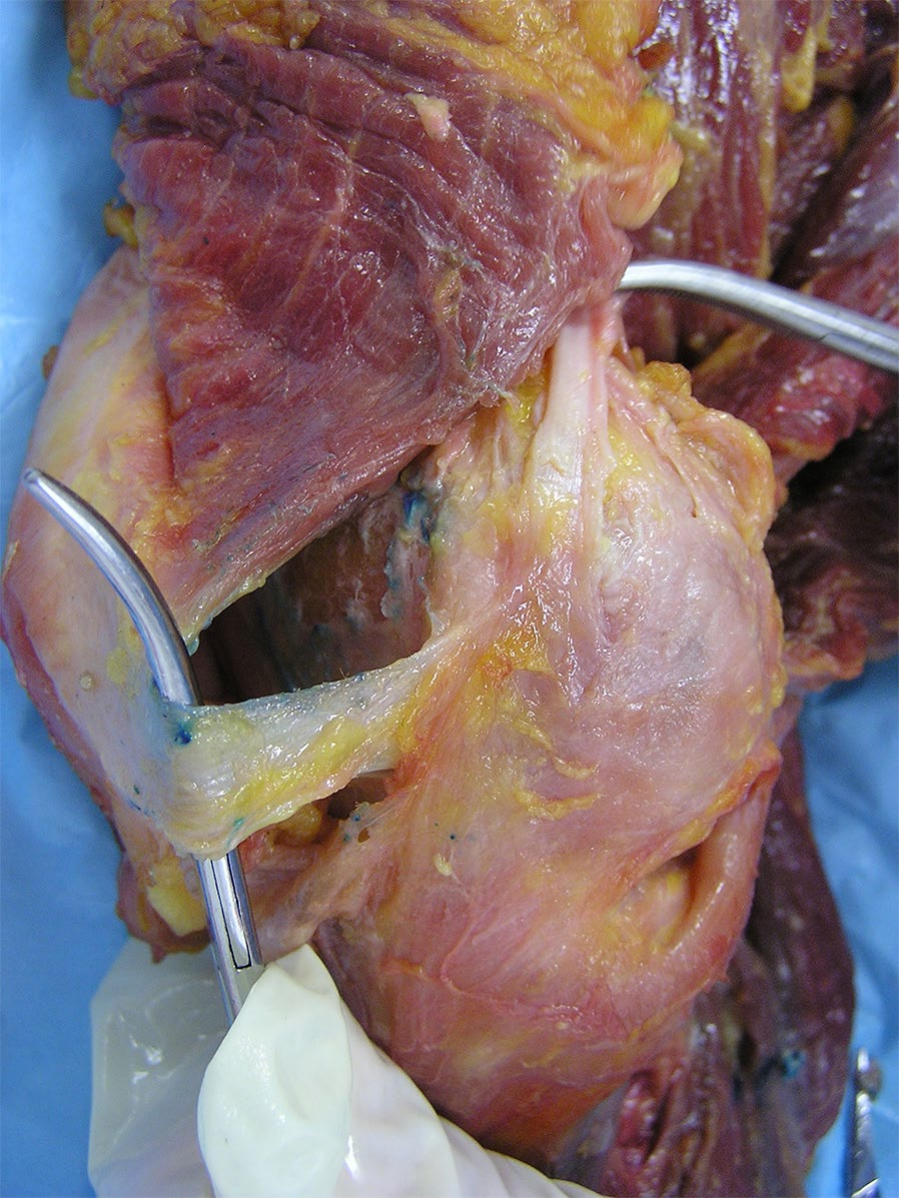
Advancing the VMO fibers distally without proper posteromedial attachment risks a more vertical pull of the VMO, rather than a posteromedial pull intended to help patella tracking

Near the MQTFL, anatomic descriptions of the MPFL agree on the superior border of the MPFL emanating from the fascial layer on the posterior aspect of the VMO. Therefore, the extent to which the MPFL is “uncovered” in any one specimen depends on the robustness and distal extent of the oblique VMO fibers (Fig. 4). In traumatic patella dislocation, the VMO has been shown to be torn from the adductor tendon, which can result in a more vertical orientation of the VMO fibers. Andrew Amis has commented on the importance of reestablishing the posterior attachments of the VMO to the adductor tendon in order to restore proper VMO fiber orientation (personal communication) (Fig. 5). If one advances the VMO fibers distally without proper posteromedial attachment, one risks a more vertical pull of the VMO, rather than a posteromedial pull intended to help patella tracking (Fig. 6).

## MPFL reconstruction

Since its very first description in 1992 by Ellera Gomes et al. [30], MPFL reconstruction has been growing in popularity [31]. Currently, MPFL reconstruction is the cornerstone of surgical treatment of recurrent lateral patellar dislocation [32]. MPFL reconstruction is routinely performed as an isolated or associated procedure that is recognized as safe, reproducible, and effective.

The clinical scenario in which isolated MPFL reconstruction is sufficient to stabilize the patella, without any bony work to compensate or a shallow trochlea, patella alta, or lateralized tibial tubercle, has not yet been categorized with clarity [15]. The original “menu à la carte,” correcting each risk factor when excessive, has been challenged with the current inclusion of MPFL reconstruction in the surgical armamentarium, as reviewed elsewhere [31].

The most widely varied aspects of MPFL reconstruction are:

- Graft choice
- Graft fixation
- Controlling graft length and its change through the knee arc of motion, which is associated with knee flexion angle at the time of graft fixation and the degree of tautness at the time of graft fixation

### Graft choice

Graft choice is an important consideration before MPFL reconstruction. Since the native MPFL has a failure load of approximately 200 N (208 N according to Mountney et al. [33]; 178 N according to LaPrade et al. [34]), the optimal graft should have similar biomechanical properties. The most popular option is autograft, such as gracilis, semitendinosus, quadriceps, quadriceps tendon, and adductor magnus tendons [35–38]. Noyes et al. [39] reported the maximum failure load for the gracilis, semitendinosus, and quadriceps tendon to be 838 N, 1216 N, and 266 N, respectively. All of these options provide higher failure loads than the native MPFL and, therefore, would be appropriate for reconstruction. Depending on availability, allogenic tissue, typically a hamstring graft, can be used. Least common is the use of synthetic grafts [40, 41].

Autografts provide good results and versatility but are affected by donor-site morbidity and graft availability, i.e., previous autograft hamstring anterior cruciate ligament (ACL) reconstruction. Moreover, in very specific conditions such as connective tissue disorders (i.e., Ehlers–Danlos and Marfan syndromes), autografts may not provide sufficient tensile strength for effective reconstruction.

Current literature shows that graft choice does not impact the outcomes of MPFL reconstruction; the choice is made based upon surgeon preference and experience.

### Graft fixation

Femoral and patellar fixation during MPFL reconstruction represent another matter of debate, since different methods on both sides have been shown to provide good results.

#### Patellar fixation

Several types of fixation have been described, including use of anchors [42, 43] or interference screw [44, 45], creation of a two-bone tunnel [35] or a bone bridge [46], and use of a transosseous suture technique [47]. Each technique is characterized by advantages and pitfalls: Although use of bone tunnels provides a stronger fixation force [48, 49], it is also associated with a higher risk of patellar fracture [50, 51]. Patellar fixation with screws or anchors can cause pain and irritation at the insertion site, with one review reporting 1.1 % of patients needing hardware removal [51]. The bone bridge technique showed a maximum load to failure inferior to that of the natural MPFL [52]. Concerning redislocation rate, conflicting results have been published when different techniques were analyzed [51, 53].

Current clinical and biomechanical studies do not identify the best fixation method on the patellar side. For this reason, the patellar fixation method is based on surgeon preference. High-level comparative studies are needed to determine which type of fixation provides the best functional results and lowest complication rates for various patient groups, patella size, and bone quality.

**Fig. 7:**
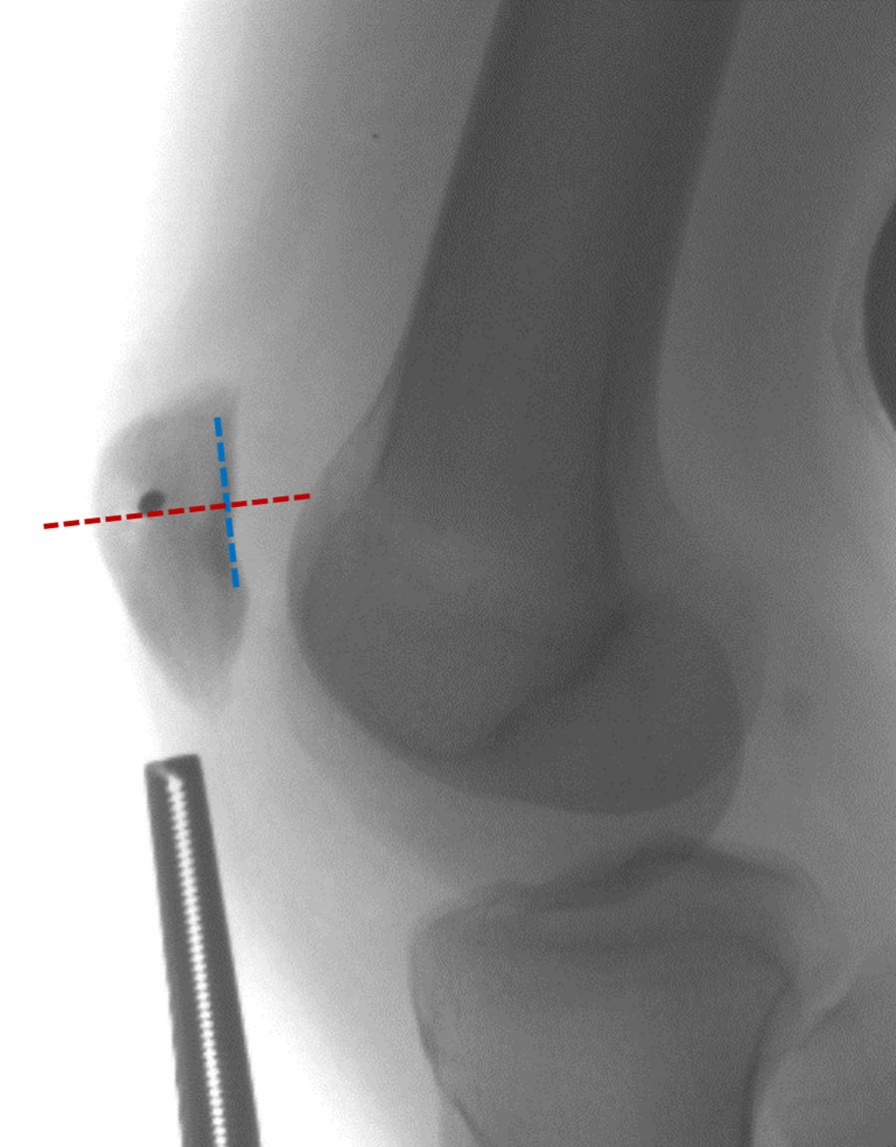
The MPFL insertion in the sagittal plane should be in the superior 50 % length of the patella cartilaginous surface

However, the location of MPFL graft fixation on the patella is important. Depending on the fixation technique and patella size, inferior graft placement can create increased distal restraint that could be a cause of reduced knee motion and/or pain in deeper knee flexion. For anatomic reconstruction, the center of the graft fixation should be located at the superomedial aspect of the patella, 27 % from the superior pole (if the distance between the superior and inferior poles is 100 %) [22]. The MPFL insertion on the patella is broad, described as between 6.1 and 23.1 mm from the superior pole [26]. Radiographically, the patellar insertion is located 7.4 mm anterior to the posterior patellar cortical line and 5.4 mm distal to the perpendicular line intersecting the proximal margin of the patellar articular surface [54]. In literature to date, measurements of the MPFL patellar location have been on the dorsal bony surface. In patellar anatomy with a long distal noncartilaginous portion (the patellar nose), use of the dorsal surface as a measurement guide could lead to poor patellar location, leading to potential pain and/or reduced knee flexion (Fig. 7). Shea et al. investigated the MPFL patellar attachment in pediatric knees and found the attachment to be 4.7 mm superior to the midline of the patella [29]. Although the anatomic location has been well described in literature, its placement appears to be less important than the placement on the femur.

#### Femoral fixation

Femoral fixation methods during MPFL reconstruction can be divided into two basic categories: bone fixation and soft tissue fixation.

Bone tissue fixation still represents the most common technique. Considering this type of fixation, the most frequent method is to create a blind bone tunnel at the femoral anatomical insertion, through which the graft is passed and fixed with an interference screw. Reliable alternatives to this kind of fixation are to secure the graft to the femur by using suspensory loop cortical button [55] or suture anchors [56]. Several studies have reported successful clinical and functional outcomes with the use of femoral tunnel and interference screw [15, 57, 58], with the most widely used technique in published studies being on the femoral side. There are only preliminary published data and descriptions of surgical technique regarding use of cortical button and suture anchors on the femoral side.

Though the first MPFL technique published by Ellera Gomes et al. [30] utilized soft tissue fixation, soft tissue fixation is most used for children and adolescents in order to avoid injury to the open growth plate. Despite its use primarily in children, good results are achieved with soft tissue technique in the adult population. The most common techniques for this kind of femoral fixation include the adductor magnus tenodesis, originally described by Avikainen et al. [59], which uses the adductor sling technique [60], or the medial collateral ligament sling technique [61], the Chassaing technique [62], and the “basket weave” technique as described by Kodkani et al. [63]. Several studies have reported good clinical and functional results using these techniques in the skeletally immature [64–66], but a higher redislocation rate when soft tissue fixation is compared with bone fixation [64, 66–68]. Soft tissue femoral fixation represents a reliable option in skeletally immature patients.

**Fig. 8:**
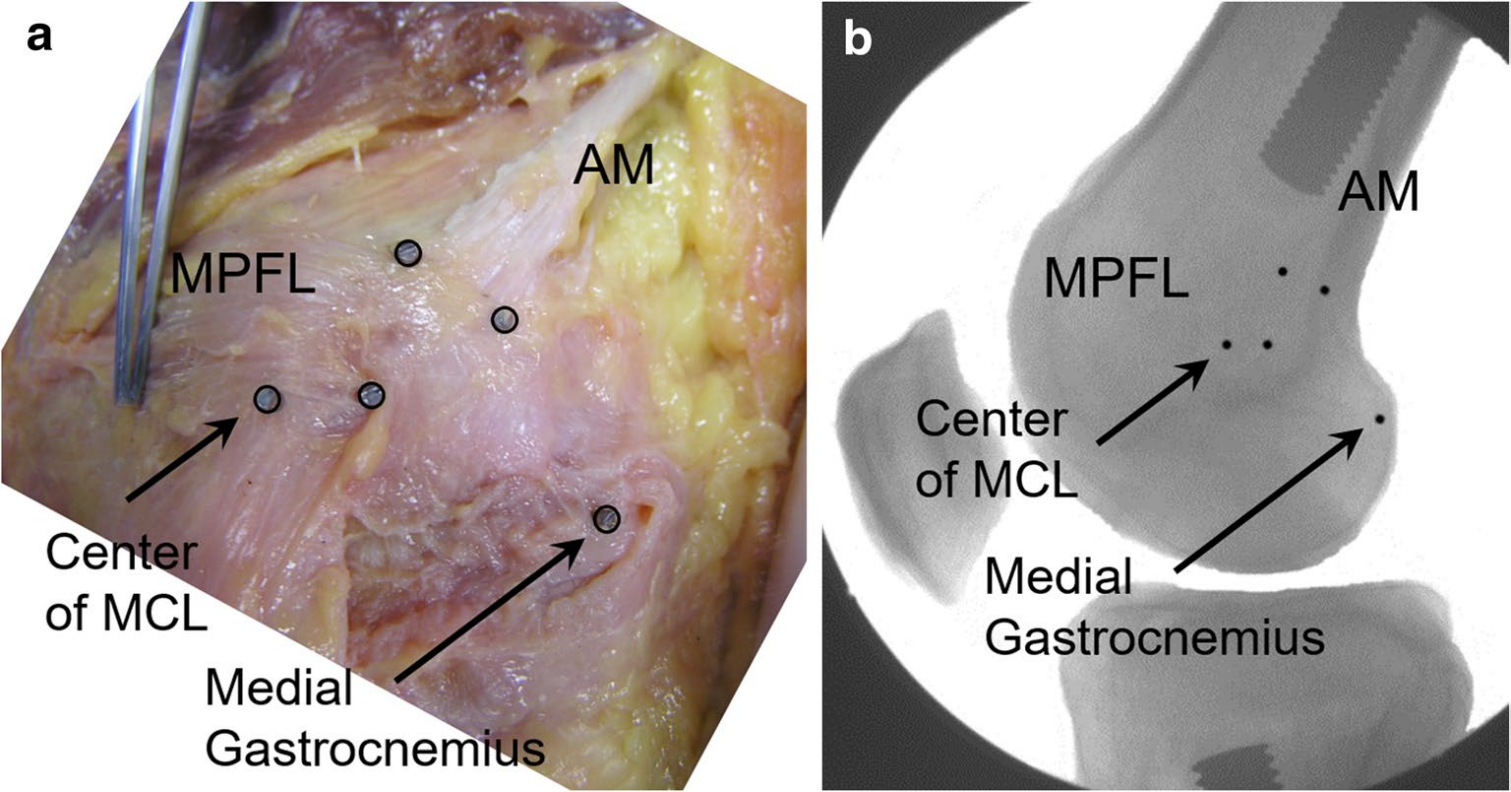
MPFL attachment sites with radiographic correlation. **a** Cadaveric specimen with metal pins outlining the MPFL and the adductor magnus (AM) tendon. **b** Radiograph of the marked cadaveric anatomic specimen

**Fig. 9:**
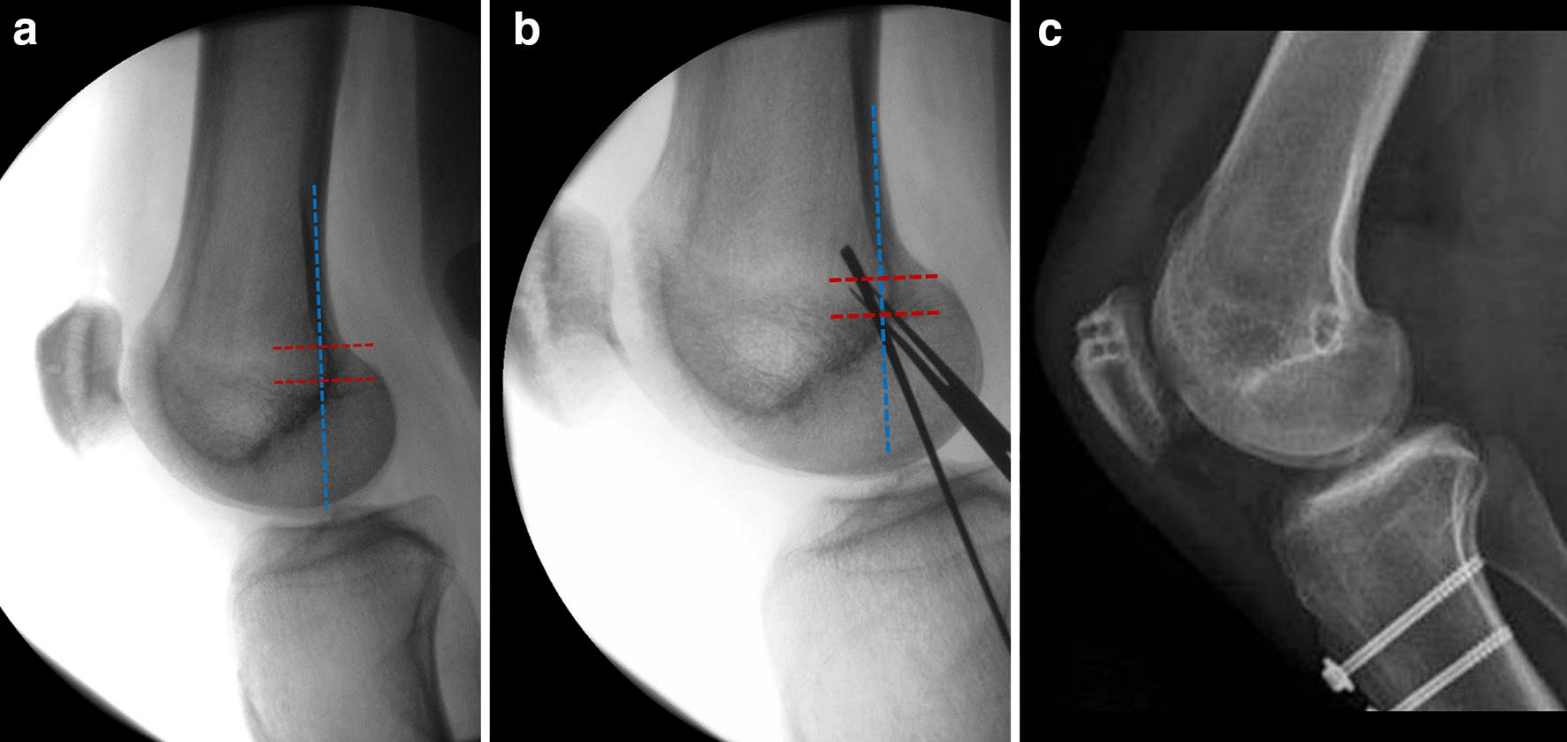
**a** Lateral radiograph of a knee with “Schöttle’s lines” in place. Radiographically, this is 1 mm anterior to the posterior femoral cortical line, 2.5 mm distal to the posterior origin of the medial femoral condyle, and proximal to the level of the posterior point of the Blumensaat line. **b** Lateral radiograph of the beath pin in place intraoperatively at the location of “Schöttle’s point.” **c** Correct position of femoral tunnel

Regardless of the technique used, the femoral location is critical to surgical success. Improper placement of the femoral tunnel may lead to altered patellofemoral biomechanics and, thus, poorer outcomes. The correct location can be found using radiographic or anatomic landmarks (Fig. 8). Stephen et al. investigated the influence of tunnel placement on patellofemoral contact pressures and biomechanics [69]. Significantly elevated medial contact pressures and medial tilt resulted from too proximal or too distal femoral tunnel positions. These findings have been supported by additional studies showing that proximal tunnel placement overloads the medial patellofemoral compartment, while a tunnel placed too distally would result in a loose, nonfunctional graft [70]. The most popular method of radiographic correlation is using “Schöttle’s point” [71] (Fig. 9), which can be located in the operating room using fluoroscopy and a true lateral radiograph.

### Knee flexion angle during fixation

The flexion angle during graft fixation helps determine the “tautness” of the graft with knee motion, and is variable in practice, being highly surgeon dependent. The debate centers on whether one believes in MPFL isometry through knee flexion arc with proper femoral fixation. All are in agreement that we need a fixation location and a ligament “tautness” that does not get tight or stretched in flexion, yet maintains an appropriate lateral restraint in early flexion.

Different studies have reported several knee flexion angles, including 20° [37], 30° [44, 72], 45° [73], 60° [46, 74], and 70° [75]. Based on a previous biomechanical study, Schöttle et al. [71] suggested in 2007 repairing the graft with the knee at 30° of flexion, since in this position the natural MPFL has its maximal restraint against patellar lateralization [21]. However, Stephen et al. [76] could not identify significant differences in patellofemoral contact when changing the knee flexion angle between 0° and 60° during graft fixation. In a recent cadaveric study, Lorbach et al. [77] concluded that fixation at 60° of knee flexion represents the position that most closely restores natural patellofemoral contact pressure, even though flexion angle did not have a significant impact on overall patellofemoral contact pressure.

### Graft tension during fixation

Tension means the state of being stretched (noun) or applying a force to something that tends to stretch it (verb). Graft tensioning is of paramount importance during graft fixation, and excessive tension should be avoided since it could be associated with stiffness, overcorrection of the patellar tilt, and early progressive degeneration of the medial patellofemoral joint. Though graft tension and the knee flexion angle at time of fixation are related, they are not synonymous. Proper graft tension, and how to achieve this during fixation, remains debatable.

In a cadaveric study, Philippot et al. [78] stated that the ideal graft tension was 10 N, and the results of this study were implemented in a clinical trial conducted by Carnesecchi et al. [79], who demonstrated excellent clinical results in 50 patients whose MPFL graft was fixed with tension of 10 N. However, a more recent biomechanical study by Stephen et al. [69] confirming the results previously obtained by Beck et al. [80] stated that a tension of 2 N in an anatomically positioned MPFL graft is sufficient to restore patellar tracking and patellofemoral joint contact pressures to the intact state, whereas tension of 10 N or greater increases medial contact pressure along with medial tilt and translation, particularly when fixation is performed in full extension.

#### Postoperative protocol

The immediate postoperative goal after MPFL reconstruction is to reduce swelling and pain. The goals in the rehabilitative phase are to eliminate pain, regain range of knee motion, and restore quadriceps strength. Postoperative protection of the knee is somewhat dependent on the strength of the graft fixation, but for most current surgical methods, progression is based on functional goals and not time.

The training phase involves regaining strength and normal functional gait pattern. For MPFL reconstruction, this is variable but typically completed by 4–6 weeks. The advanced training phase involves examination of complex body movement patterns, primarily to reduce the risk of reinjury and maximize surgical success. This phase is typically 6–12 weeks postoperatively.

Regarding return to sport, the injured patient is often eager for an expected timeline for return to full function. Estimates can be given, but the athlete and physician must be aware that the ultimate return to activities, be they daily activities or higher-level competition, depends on recovery of strength, flexibility, and appropriate body movement patterns.

Various functional tests, more commonly used in knee ligament surgery, can be employed to aid estimation of patient readiness to return to activities. The average return to sport, depending on patient- and sport-specific factors, is 4–6 months.

## Conclusions

An explosion of knowledge based on PF anatomy and biomechanics principles has positively influenced treatment of PF injuries, in particular lateral patellar dislocation. Patella stabilization surgery demands broad knowledge of anatomic and biomechanical principles as reviewed herein.

## Abbreviations

MCL: medial collateral ligament
MPFL: medial patellofemoral ligament
MPML: medial patellomeniscal ligament
MPTL: medial patellotibial ligament
MQTFL: medial quadriceps tendon-femoral ligament
PF: patellofemoral
VMO: vastus medialis obliquus muscle
